# First-in-Human Assessment of Gut Permeability in Crohn’s Disease Patients Using Fluorophore Technology

**DOI:** 10.1016/j.gastha.2024.02.003

**Published:** 2024

**Authors:** Lori R. Holtz, B. Darren Nix, Sewuese E. Akuse, Carla Hall-Moore, Rodney D. Newberry, Matthew A. Ciorba, Parakkal Deepak, Maria Zulfiqar, Jeng-Jong Shieh, James R. Johnson, I. Rochelle Riley, Richard B. Dorshow

**Affiliations:** 1 Department of Pediatrics, Washington University School of Medicine, St. Louis, Missouri; 2 Department of Internal Medicine, Washington University School of Medicine, St. Louis, Missouri; 3 Department of Radiology, Washington University School of Medicine, St. Louis, Missouri; 4 MediBeacon Inc., St Louis, Missouri

**Keywords:** Crohn’s Disease, Dual Sugar Absorption Test, Fluorescence Tracer Agent, Intestinal Permeability, MB-102, Relmapirazin

## Abstract

**Background and Aims:**

The dual sugar absorption test as a classic measure of human intestinal permeability has limited clinical utility due to lengthy and cumbersome urine collection, assay variability, and long turnaround. We aimed to determine if the orally administered fluorophore MB-102 (relmapirazin) (molecular weight [MW] = 372) compares to lactulose (L) (MW = 342) and rhamnose (R) (MW = 164)-based dual sugar absorption test as a measure of gut permeability in people with a spectrum of permeability including those with Crohn’s disease (CD).

**Methods:**

We performed a single-center, randomized, open-label, crossover study comparing orally administered MB-102 (1.5 or 3.0 mg/kg) to L (1000 mg) and R (200 mg). Adults with active small bowel CD on magnetic resonance enterography (cases) and healthy adults (controls) were randomized to receive either MB-102 or L and R on study day 1, and the other tracer 3 to 7 days later. Urine was collected at baseline and 1, 2, 4, 6, 8, 10, and 12 hours after tracer ingestion to calculate the cumulative urinary percent excretion of MB-102 and L and R.

**Results:**

Nine cases and 10 controls completed the study without serious adverse events. Urinary recovery of administered MB-102 correlated with recovery of lactulose (r-squared = 0.83) for all participants. MB-102 urine recovery was also tracked with the L:R ratio urine recovery (r-squared = 0.57). In controls, the percentages of L and MB-102 recovered were similar within a narrow range, unlike in CD patients.

**Conclusion:**

This first-in-human study of an orally administered fluorophore to quantify gastrointestinal permeability in adults with CD demonstrates that MB-102 is well tolerated, and its recovery in urine mirrors that of percent L and the L:R ratio.

## Introduction

Maintenance of epithelial integrity is a cardinal function of the gastrointestinal tract because the mucosal barrier restricts the passage of microbes and antigens from the gut lumen into the systemic circulation. Increased intestinal permeability, colloquially termed “leaky gut,” occurs when this barrier is damaged, principally by disruption of tight junctions between cells. Increased permeability is a common feature of many gut inflammatory processes and is also postulated to play a role in extra-intestinal disorders.^1^

The dual sugar absorption test (DSAT) is the classic functional and most direct measure of human intestinal permeability.^2^ This test involves an oral challenge with a solution of 2 sugars and the collection of urine over several hours to determine the extent to which the sugars are absorbed by the host gut. In its most common iteration, the DSAT employs a disaccharide (usually lactulose, L, molecular weight [MW] = 342) and a monosaccharide, traditionally mannitol (M) (MW = 182) or rhamnose (R) (MW = 164). R is increasingly used because M can be found in urine after an overnight fast,^3^ probably from dietary additives. However, the choice, concentrations, and volumes of the sugar solutions, timing of urine collection, and methods of assay vary considerably. The test also has technical challenges related to the processing of the urine and assay variability. These limitations diminish the appeal of the DSAT for widespread clinical use.

Intestinal permeability is an appealing functional test that could aid in the diagnosis of Crohn’s disease (CD) and response to treatment. Multiple studies report that patients with CD have greater intestinal permeability^4^, ^5^, ^6^, ^7^ than healthy controls. Recent data demonstrate that increased intestinal permeability is associated with the subsequent development of CD among genetically susceptible individuals.^8^ Proteomics signatures suggest loss of barrier function is common in the earliest stages of CD.^9^ Finally, barrier healing is associated with decreased risk of disease progression in patients with inflammatory bowel disease in remission, and permeability is superior to endoscopic and histologic remission in predicting risk.^10^ However, intestinal permeability testing is rarely used in clinical care of CD. The lack of an easy test of human intestinal permeability is no doubt a major impediment to adoption of this approach. The use of orally ingested fluorophores, which can be measured either in the urine or transdermally, might overcome the technical limitations of the DSATs.

Preclinical models suggest that orally administered fluorescent tracer agents can be used to measure gut permeability. First, urinary recovery of pyrazine fluorophores MB-402 (MW = 422) and MB-301 (MW = 198) closely approximates the urinary recovery of L and R after oral gavage in an indomethacin injury rat model.^11^ L and R and MB-402 and MB-301 both reflect small bowel injury in an indomethacin dose-dependent manner. In a subsequent study of transdermal detection of extraintestinal fluorophores MB-404 (MW = 492) and MB-301, the uptake of the heavier tracer was unequivocally greater in the injured than in the control rats.^12^ In this study, it was only the larger fluorophore that actually distinguished the injured from control rats, suggesting that only one fluorophore might be needed to distinguish diseased from normal intestinal permeability.^12^ Indeed, lactulose urinary clearance is advocated in human studies over the disaccharide to monosaccharide ratio.^13^ Properties of fluorophores and sugars are summarized in Table A1.

Here we report a randomized, open-label, crossover trial of orally administered tracers MB-102 and L and R (ClinicalTrials.gov NCT03962998). We chose MB-102 because of its equivalency to paired fluorophores and is already in clinical studies for transdermal measurement of glomerular filtration rate (GFR) after intravenous injection^14^, ^15^, ^16^ and thus amenable to another indication, ie, measuring gut permeability. To compare MB-102 and DSAT clearances over broad ranges, we enrolled healthy adults as controls and adults with evidence of active small bowel CD on magnetic resonance enterography (MRE) as cases. Here we present the results of this first-in-human study comparing the recovery in urine of orally administered MB-102 to DSAT in people with CD and volunteers without known gut inflammation. This study is a prelude to testing gut permeability by noninvasive transdermal fluorescence measurement of MB-102.

## Methods and Materials

### Compounds

*MB-102 (INN designation relmapirazin)* (supplied by MediBeacon Inc, St. Louis, MO, USA, as a sterile solution of 18.6 mg/mL in phosphate-buffered saline) is a bis-serine aminopyrazine currently used as a fluorescent GFR tracer agent in clinical studies.^17^ It is not metabolized in vivo, has negligible protein binding, is chemically and photometrically stable, is cleared from the circulation by glomerular filtration without tubular secretion or absorption (as are L, R, and M), and can be easily detected in plasma and urine by high performance liquid chromatography. MB-102 has a peak excitation wavelength of 440 nm and a peak emission wavelength of 560 nm.^18^ Twenty-four formal nonclinical tests of MB-102 have shown no toxicologic concerns.^19^, ^20^, ^21^, ^22^ In the clinical studies for GFR determination (NCT02098187, NCT02098174, NCT02772276, and NCT05425719), over 500 participants have been dosed intravenously without serious adverse events.

#### Lactulose

Lactulose was obtained from Cumberland Pharmaceuticals, Nashville, TN, USA, as dry powder.

#### Rhamnose

L-rhamnose was obtained from TCI, Portland, OR, USA, as dry powder.

### Study Design and Participants

This single-center, randomized, open-label, crossover study was approved by the Washington University in St. Louis Human Research Protection Office and conducted per Good Clinical Practice guidelines with US Food and Drug Administration approval (IND 139090). The study was registered at www.clinicaltrials.gov as NCT03962998.

All eligible participants had estimated GFRs greater than 60 ml/min/1.73 m^2^ (as determined by the Chronic Kidney Disease Epidemiology Collaboration equation^23^) and agreed to not use nonsteroidal anti-inflammatory drugs until study completion. Cases were adults with active small bowel CD on MRE confirmed by an abdominal radiologist, characterized by mural hyperenhancement and/or bowel wall thickening with engorged vasa recta within 45 days of the screening visit. Inclusion/exclusion criteria for cases and controls are provided in Table 1.

**Table 1:** Eligibility Criteria

All participants: Inclusion criteria	All participants: Exclusion criteria
Age: >18 y; males, nonpregnant, or lactating females willing to consent and comply with study requirements	Pregnant, lactating females, or individuals unwilling to use adequate methods of birth control
Normal or nonclinically significant baseline ECG	Participation in other interventional trials within 30 d of dosing
eGFR > 60 ml/min/1.73m^2^	Unable to tolerate an overnight fast
Agreement to not use NSAIDs until study completion	NSAID use within 14 days of day 1
	History of drug or alcohol abuse; diagnosis of ulcerative colitis, indeterminate colitis, pseudomembranous colitis, or celiac disease; prior GI surgery within 12 wk prior to screening; type 1 or 2 diabetes; history of severe hypersensitivity or anaphylactoid reactions; known history of HIV or AIDs; current urinary tract infection; BMI >35; prior exposure to relmapirazin
	Changes to medications between testing day 1 and testing day 2
Additional exclusions for normal participants	
Fecal transplant within 1 y; prior or current history of graft-vs-host disease, diverticulitis, fatty liver, Crohn’s disease, an auto-immune disease, small bowel malignancy, or resection surgery. Undiagnosed chronic gastrointestinal upset, food intolerance history, or recent history of significant unplanned weight loss, blood in the stool, or acute episodes of diarrhea	
Current use of any biologic therapy or any of the following medications: Sulfasalazine, mesalamine, olsalazine, balsalazide, prednisone, cyclosporine, azathioprine, 6-mercaptopurine, tacrolimus, methotrexate, IVIG, antidiarrheal agents	
First-degree relative (sibling, parent, child) has inflammatory bowel disease (Crohn’s disease, ulcerative colitis, proctitis, indeterminate colitis).	
Additional inclusion for Crohn’s disease participants	
Participants with active small bowel Crohn’s disease recently diagnosed or undergoing ongoing evaluation who have an abnormal magnetic resonance enterography (MRE) within 45 d prior to screening.	
Active Crohn’s disease must be characterized by mucosal hyperemia and/or bowel wall thickening and/or vascular engorgement.	
Additional exclusions for Crohn’s disease participants	
Participants on total parenteral nutrition (TPN)	
Participants with greater than 25% of their estimated small bowel length were resected. Colectomies are allowed.	

ECG, electrocardiogram; eGFR, estimated glomerular filtration rate; IVIG, intravenous immunoglobulin; NSAID, non-steroidal anti-inflammatory drugs.

Participants underwent a screening visit, at which time laboratory tests were obtained and an electrocardiogram and physical examination were performed. They returned within 30 days for the first test of permeability (test day 1), where they were randomly assigned to receive either MB-102 or the sugars. This was followed by the other tracer 3 to 7 days later for the second test of permeability (test day 2). Participants returned for a follow-up visit between 4 and 10 days after the second test (Table 2).

**Table 2:** Study Activities

Data obtained	Screening	Test day 1 Sugar testing^a^	Test day 2 MB-102^a^	Follow-up visit
	≤30 d prior to test day 1		3 to 7 d following test day 1	7 d ± 3 d after day 2
Informed consent	X			
Inclusion/exclusion	X	X		
Demographics	X			
Medical history	X	X		
Pregnancy test for WOCBP	X		X	
Physical exam or limited physical assessment	X	X	X	X
Vital signs	X	X	X	X
Height and weight	X			
Clinical labs	X	X	X	X
Administration of dual sugar test		X		
Administration of MB-102			X	
Urine collection		X	X	
ECG	X	X	X	
Concomitant therapies	X	X	X	X
Adverse events		X	X	X

Clinical labs = screening day: complete metabolic panel (CMP), phosphorus, total and direct bilirubin, uric acid, PT/INR aPTT, complete blood count (CBC) with differential, and urinalysis. Test days 1 and 2 (obtained predose and 12 h postdose): CMP, phosphorus, total and direct bilirubin, uric acid; CBC, with differential, and urinalysis.

ECG: performed predose and 12 h postdose on test days 1 and 2.

aPTT, activated partial thromboplastin time; ECG, electrocardiogram; INR, international normalized ratio; PT, prothrombin time; WOCBP, women of childbearing potential.

a Participants randomized to determine if they received dual sugar or MB-102 on test day 1.

### Administration of Dual Sugar Test or MB-102

The DSAT solution consisted of 200 mg of rhamnose and 1000 mg of lactulose in 10 mL of sterile water. MB-102 solution for administration was dosed at 1.5 or 3.0 mg/kg (4 or 8 μmol/kg) of body weight. Both solutions were prepared by the Washington University Research Pharmacy. Participants fasted except for non-fat liquids for 12 hours before and 2 hours after oral administration of either tracer. Ingestion of either tracer was followed immediately by 300 mL of water. Standardized meals were provided on both test days.

### Collection and Analysis of Urine

On each test day, urine was collected predose and 1, 2, 4, 6, 8, 10, and 12 hours postdose. Urine was also collected at all unscheduled voids postdose. Urine volumes were recorded for every void. Samples were aliquoted and immediately frozen at −80 °C until analyzed for agent concentration. MB-102 concentration in urine was measured using a ultra-performance liquid chromatography system with fluorescence detection, as previously reported.^24^

Lactulose and rhamnose concentrations in urine were quantified using normal-phase isocratic high performance liquid chromatography-tandem electrospray ionization mass spectrometry at the Mayo Clinic, as described.^25^

### Scoring of Crohn’s Disease Activity on MRE

The degree of activity of small bowel CD on MRE was quantified using the simplified magnetic resonance index of activity (MaRIAs) score for ileocolonic disease.^26^ The MaRIAs score was calculated for 6 defined anatomic regions including distal ileum, ascending colon, transverse colon, descending colon, sigmoid colon, and rectum by an abdominal radiologist as follows: (1x wall thickness >3 mm) + (1x mural edema) + (1x fat stranding) + (2x ulceration). Inflammatory mural edema was defined as high signal intensity on T2-weighted MR sequences with or without fat saturation, compared to normal-appearing bowel loops. Fat stranding was defined as loss of normal sharp interface between the intestinal wall and mesentery due to edema or fluid in the perienteric fat of the involved segment. Mucosal ulceration was considered present when a discrete defect in the bowel mucosa was identified primarily using T2-weighted sequences or postcontrast volumetric interpolated breath-hold examination MRI sequences. The MaRIAs score for each of the 6 segments were then added to compute the global MaRIAs score for each patient.

### Statistics

Linear regression analysis (slope and r-squared) was used to relate urinary clearance of MB-102 and lactulose, or L:R. Secondary correlation analysis was performed using Spearman's r. The significance of differences between continuous and categorical variables was determined by the Mann-Whitney and Fisher's exact tests, respectively. All *P*-values were two-sided; α-values <0.05 were considered statistically significant. The Šídák-Bonferroni method was used to correct for multiple comparisons. Analyses were performed with Prism 9.3.1 (GraphPad).

## Results

### Demographics and Tracer Tolerance

**Figure 1:**
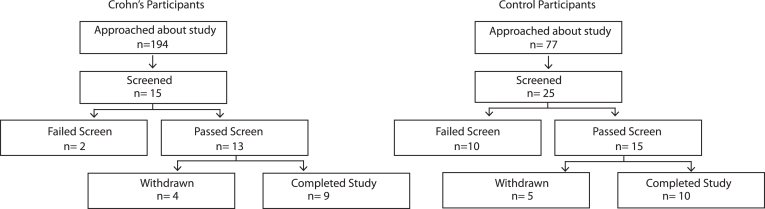
Participant recruitment flow chart.

Figure 1 demonstrates the recruitment flow, and Table 2 provides the study procedures for the 9 cases and 10 controls completing both tracer studies. The median age (interquartile range) of the cases was 36 (33, 37) years, and that of the controls was 50 (35, 58). Four (44%) cases and 5 (50%) controls were female. All cases and 9 (90%) controls were Caucasian. Seven participants (5 controls and 2 CD) received 1.5 mg/kg of MB-102, and the remaining 12 participants received 3.0 mg/kg of MB-102 (Figure A1). The orally administered MB-102 was well-tolerated, with no serious adverse events.

### Crohn’s Disease Activity

**Figure 2:**
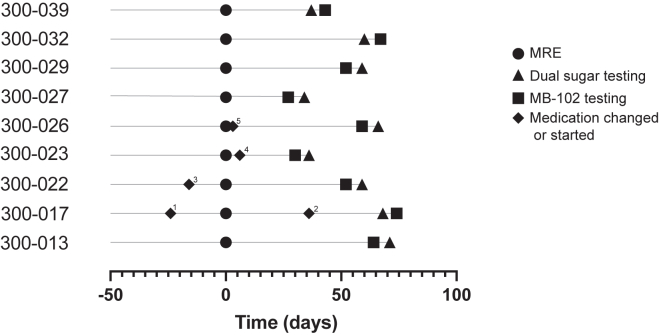
Timeline for Crohn’s disease participants showing timing of MRE, permeability testing, and medication changes or initiation in proximity to the trial. ^1^Methotrexate initiated. ^2^Increased frequency of biologic. ^3^Biologic initiated. ^4^Azathioprine initiated. ^5^Antibiotic therapy started for fistulizing disease.

All cases had active small bowel CD by MRE (Figure 2) with median (interquartile range) distal ileum MaRIAs scores of 3 (2.5‒5). One case had undergone total colectomy and distal ileal resection 14 years earlier and had an ileostomy. Another case had undergone ileocolic resection and extended right hemicolectomy 5 years earlier. Two cases were taking no medications for their CD, and the remaining 7 were on biologic therapies at the time of their permeability testing. In response to symptoms and/or findings on MRE, 3 case participants changed medications between MRE and permeability testing. Two cases changed medications shortly before their MRE.

### Recovery of Administered Dose in the Urine for Each Agent

Prior to oral dose administration, neither lactulose nor MB-102 was detected in any participant’s urine. However, the urine of one control and 3 cases had low concentrations of rhamnose at baseline.

The cumulative recovery in urine for each agent as a function of time post administration is shown in Figure A1. In general, at the 12-hour study end, the recovery of MB-102 was still increasing while the recovery of the 2 sugars had plateaued. Among the controls, the recovery of MB-102 did not differ significantly by dose administered (Figure A2).

**Figure 3:**
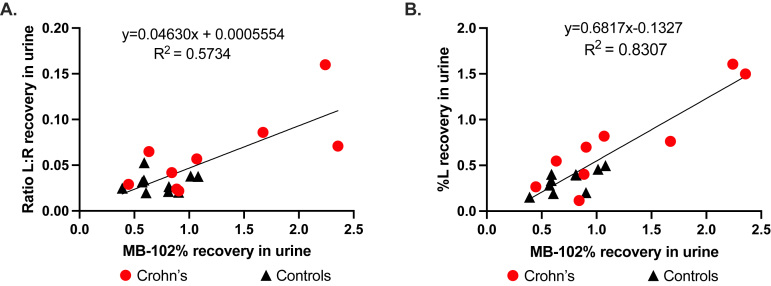
Percent of administered dose of MB-102 compared to (A) L:R (B) lactulose recovered in control participants and those with Crohn’s disease over 12 hours of collection.

The total dose recovered for MB-102 compared to the total L:R dose recovered for each participant is shown in Figure 3A. Each control has low values for each of these 2 variables. The overall correlation between MB-102 and L:R recovery is modest (linear regression R^2^ = 0.573, spearman r = 0.465, *P* = .0449).

The total dose recovered for MB-102 compared to the total lactulose dose alone recovered for each participant is shown in Figure 3B. Here, the comparison between these 2 variables tracks more closely than the comparison using L:R, especially for those with a higher degree of permeability (linear regression R^2^ = 0.831, spearman r = 0.753, *P* = .0002).

**Figure 4:**
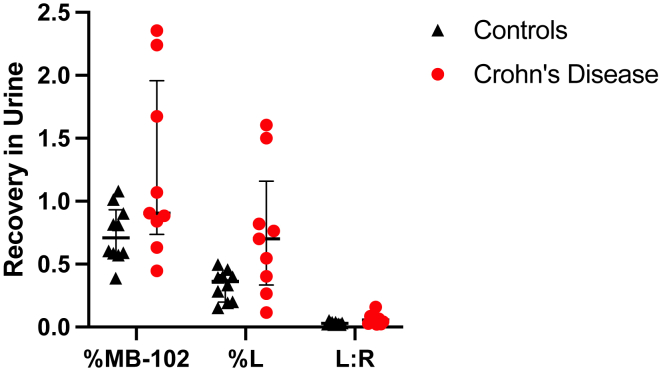
Percent recovery of MB-102, L, and L:R in control vs CD participants. Lines denote median and interquartile range.

While not the primary goal of the study, we compared the percent recovery of MB-102, L, and L:R in participants with CD and controls (Figure 4). There is a spectrum of permeability seen in the participants with CD. Furthermore, across all measures, the median permeability seen in CD exceeds that of controls. However, this difference was not statistically significant.

## Discussion

In general, the control participants had less recovery of lactulose or MB-102 in the urine compared to the participants with CD (Figure 4). The percentage of the administered dose recovered in the urine of all participants was highly correlated between MB-102 and lactulose (Figure 3B). The wide range of percent lactulose or MB-102 recovered in CD patients likely reflected the variable degree of disease activity at the time of testing. As shown in Figure 2, there was a span of time between diagnosis with MRE and the MB-102 and DSAT assay studies. Some of the cases responded to therapeutics during this interval, and some did not, thus accounting for the gradation of agent recovery in the urine. This spectrum of permeability seen in the CD patients most likely reflects disease activity, which has been noted previously in the literature.^27^

The correlation of MB-102 with L:R was not as strong as it was with percent L. Recent papers endorse using percent L excretion as an indicator of small bowel permeability over the DSAT due to confounding effects of the small-molecule sugar in the epithelium.^13^ The reported measurements herein bolster this observation.

In this first-in-human study of a pyrazine fluorophore to determine gastrointestinal permeability, no serious adverse events were observed, and oral administration of MB-102 was well tolerated by all participants.

Prior studies using concomitantly administered radioisotopes and oral sugars have suggested that urine collected 0 to 2 hours after ingestion reflects small intestinal permeability and at 8 to 24 hours reflect colonic permeability.^28^ CD participants 300-029 in this study had a colectomy and end ileostomy, allowing a unique look at the timing of small bowel permeability. Interestingly, the MB-102 and percent lactulose recovered in this participant’s urine largely plateaued at 6 hours postingestion (Figure A3) suggesting that measurements after 6 hours reflect colonic permeability.

MB-102 is currently being used in clinical studies for the transdermal measurement of the GFR after intravenous injection.^14^, ^15^, ^16^, ^17^ The ability to detect MB-102 transdermally paves the way for a specimen-free detection of this tracer after oral administration. This would avoid the lengthy and cumbersome urine collection, assay variability, and long turnaround, each of which renders the DSAT impractical for clinical care.

There are multiple specific use scenarios for intestinal permeability testing in those with confirmed or suspected inflammatory bowel disease. In CD, permeability is repaired in some patients within weeks after dosing with biologics.^29^^,^^30^ An easy-to-administer test for permeability, as envisaged by the transcutaneous detection of orally administered MB-102, could provide early indications of responsiveness and prompt a timely switch from biologics that appear ineffective in individual patients. Intestinal permeability is also potentially a differentiating characteristic in inflammatory bowel disease patients with subjective symptoms without objective findings of inflammation, in whom it is important to assess mucosal healing noninvasively.^31^ Recent reports suggest that prior to the onset of CD, genetically susceptible people had increased intestinal permeability accompanied by variant gut bacterial communities.^32^ The possibility exists that close monitoring of gut function in such individuals could lead to interventions to better manage, or prevent, the first presentation of symptomatic CD.

## Conclusion

In a first-in-human study of the pyrazine fluorophore MB-102 for gastrointestinal permeability determination, the result for all participants (controls and CD patients) was a high degree of correlation in the administered dose recovered in the urine between MB-102 and lactulose. No serious adverse effects were observed. This result paves the way for transdermal detection of MB-102 following oral administration with its advantages of noninvasive detection, measurement at the point of care, and no specimen collection.

## Supplementary Materials

Table A1

Figure A1 Cumulative urine recovery of (A) MB-102, (B) lactulose, and (C) rhamnose over time. Asterisk indicates participants receiving 1.5 mg/kg of MB-102.

Figure A2 Percent recovery of MB-102 among controls by dose administered. Lines denote mean and standard deviation.

Figure A3 Cumulative urine recovery of MB-102 (blue) and lactulose (orange) for participant 300-029 with colectomy and end ileostomy.
