# Supplementary material for: First-in-Human Assessment of Gut Permeability in Crohn’s Disease Patients Using Fluorophore Technology
Source: Gastro Hep Adv. 2024 Feb 16;3(4):491–7. doi: 10.1016/j.gastha.2024.02.003 (PMC11129951; doi:10.1016/j.gastha.2024.02.003)
Supplement: Table A1 [file mmc1.docx]

| Fluorophore Tracers | Sugar Tracers |
| --- | --- |
| 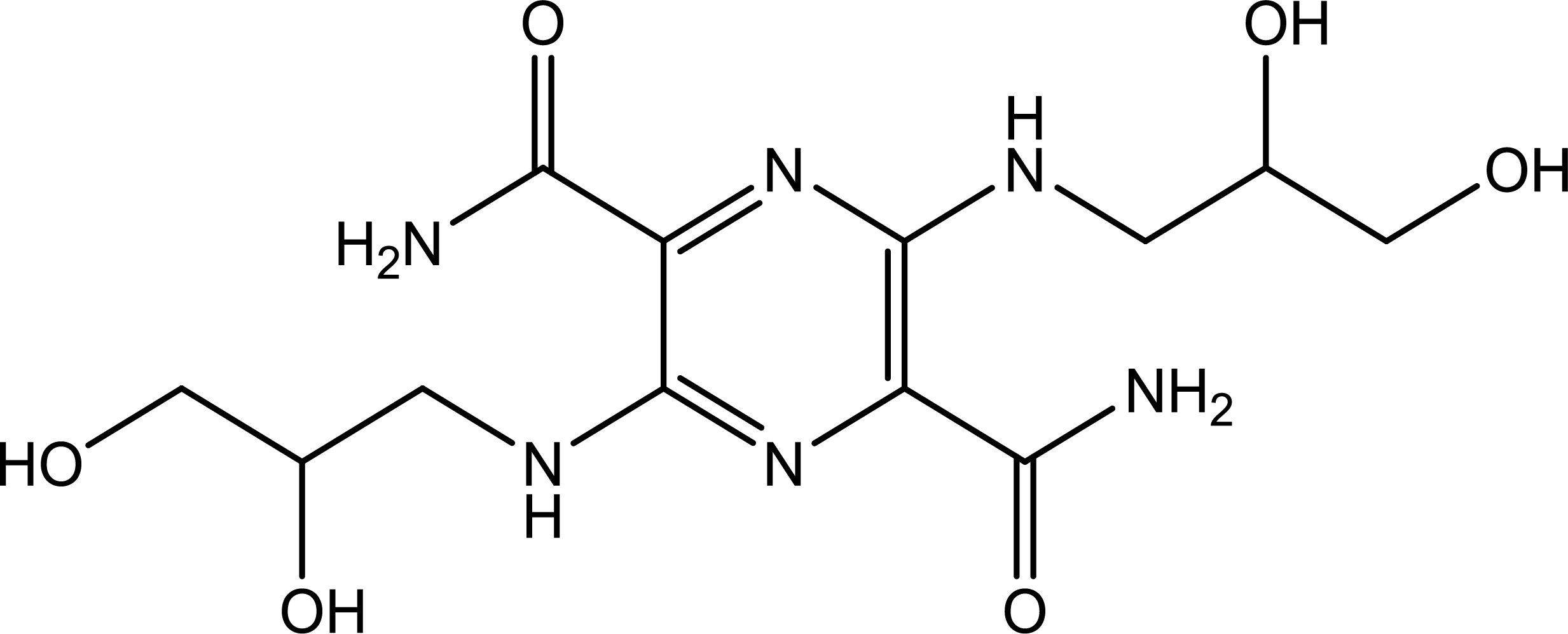  **MB-402**  MW = 422  Light absorption maximum: 500 nm  Emission maximum: 620 nm  Used in rat model ^11^. | 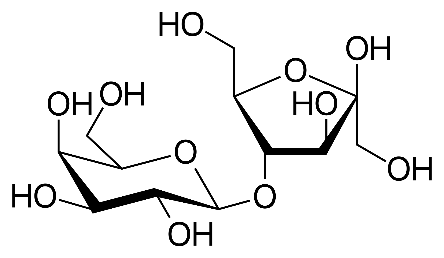  **Lactulose^1^**  MW = 342  Used in rat model ^11^ and this paper. |
|   **MB-301**  MW = 198, dose = 16 mg/kg  Light absorption maximum: 405 nm  Emission maximum: 540 nm  Used in rat model ^11, 12^ | 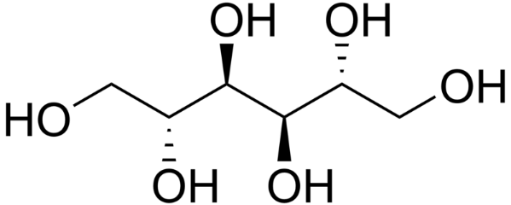  **Mannitol^2^**  MW = 182 |
|   **MB-404**  MW = 492  Light absorption maximum: 500 nm  Emission maximum: 600 nm  Used in rat model ^12^. | 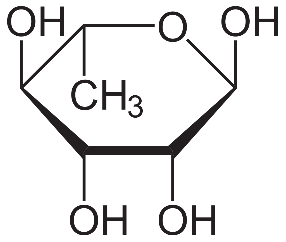  **Rhamnose^3^**  MW = 164  Used in rat model ^11^ and this paper. |
| **MB-102**  (relmapirazin)  ****  MW = 372  Light absorption maximum: 440 nm  Emission maximum: 560 nm  Described in ^18-22, 33-35^  **and** this paper. |  |
| **Supplemental Table 1. Properties of tracers discussed in this report.** Chemical structures, molecular weights (MW) and, if appropriate, light absorption and emission maxima for fluorophore and sugar tracers tested.  ^1^National Center for Biotechnology Information. PubChem Compound Summary for CID 11333, Lactulose. <https://pubchem.ncbi.nlm.nih.gov/compound/Lactulose>.  ^2^National Center for Biotechnology Information. PubChem Compound Summary for CID 6251, Mannitol. <https://pubchem.ncbi.nlm.nih.gov/compound/Mannitol>.  ^3^National Center for Biotechnology Information. PubChem Compound Summary for CID 25310, L-Rhamnose. <https://pubchem.ncbi.nlm.nih.gov/compound/L-Rhamnose>. | |
