# Supplementary figures and images for: First-in-Human Assessment of Gut Permeability in Crohn’s Disease Patients Using Fluorophore Technology
Source: Gastro Hep Adv. 2024 Feb 16;3(4):491–7. doi: 10.1016/j.gastha.2024.02.003 (PMC11129951; doi:10.1016/j.gastha.2024.02.003)

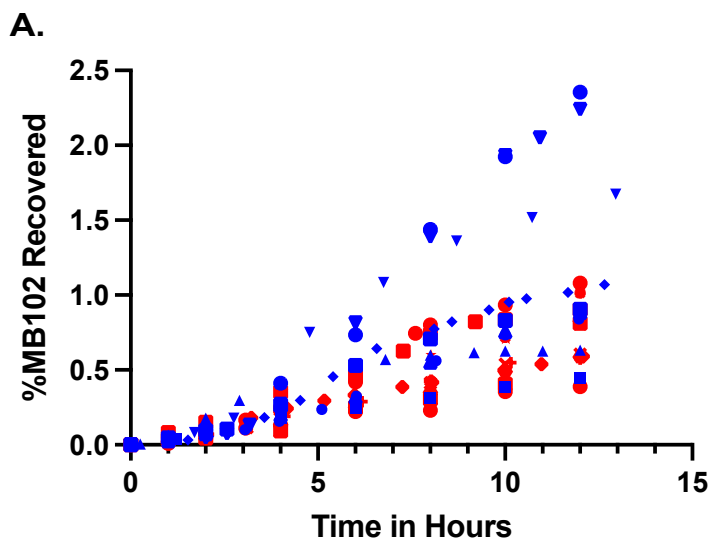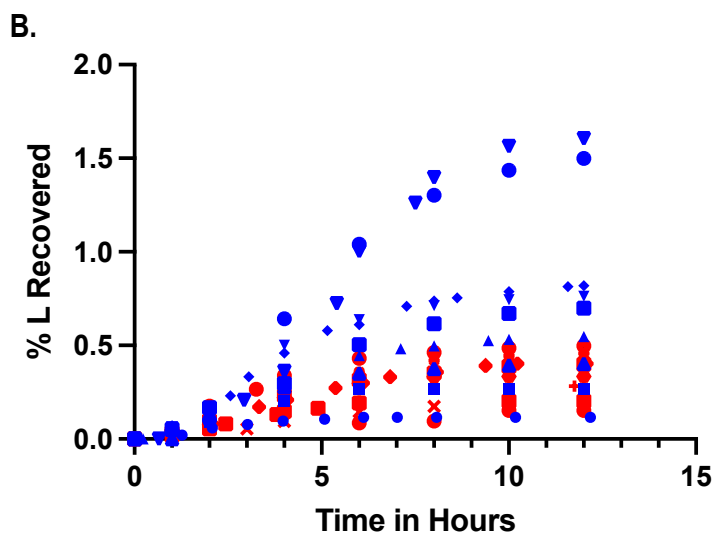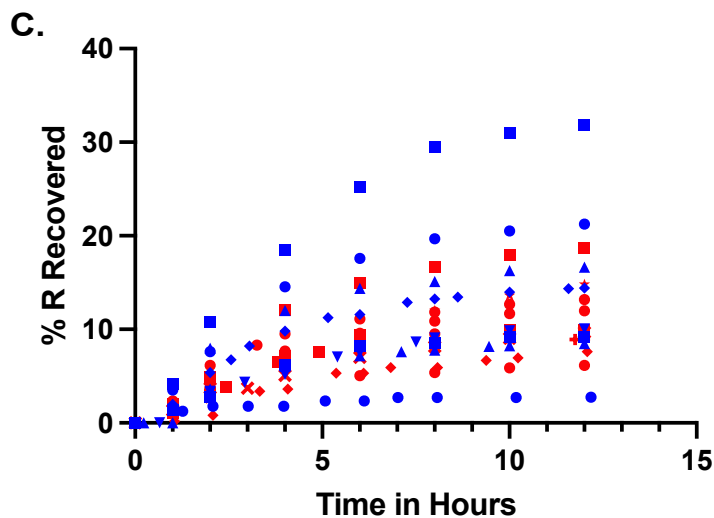

Supplement: Figure A1 — Cumulative urine recovery of (A) MB-102, (B) lactulose, and (C) rhamnose over time. Asterisk indicates participants receiving 1.5 mg/kg of MB-102. [file mmc2.pdf]

**MB-102% Recovered**

1.5  
1.0  
0.5  
0.0

ns

1.5 mg/kg

3.0 mg/kg

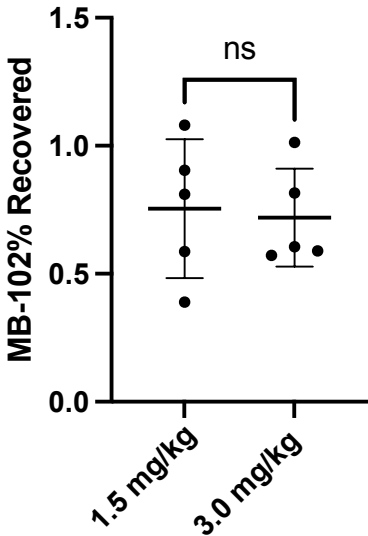

Supplement: Figure A2 — Percent recovery of MB-102 among controls by dose administered. Lines denote mean and standard deviation. [file mmc3.pdf]

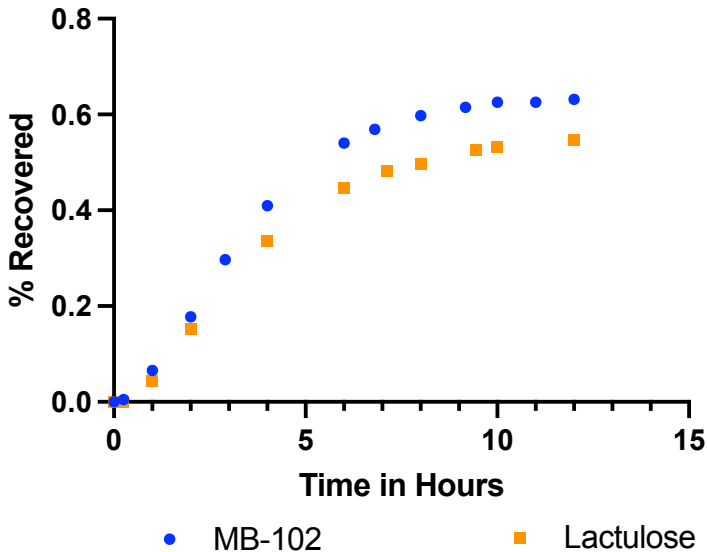

Supplement: Figure A3 — Cumulative urine recovery of MB-102 (blue) and lactulose (orange) for participant 300-029 with colectomy and end ileostomy. [file mmc4.pdf]
